# Temporal Progression of Retinal Progenitor Cell Identity: Implications in Cell Replacement Therapies

**DOI:** 10.3389/fncir.2017.00105

**Published:** 2017-12-19

**Authors:** Awais Javed, Michel Cayouette

**Affiliations:** ^1^Cellular Neurobiology Research Unit, Institut de Recherches Cliniques de Montreal (IRCM), Montreal, QC, Canada; ^2^Molecular Biology Program, Université de Montréal, Montreal, QC, Canada; ^3^Department of Medicine, Université de Montréal, Montreal, QC, Canada; ^4^Division of Experimental Medicine, Department of Anatomy and Cell Biology, McGill University, Montreal, QC, Canada

**Keywords:** cell replacement therapies, retinal degenerative diseases, temporal identity, neural progenitors, cone photoreceptors, stem cells

## Abstract

Retinal degenerative diseases, which lead to the death of rod and cone photoreceptor cells, are the leading cause of inherited vision loss worldwide. Induced pluripotent or embryonic stem cells (iPSCs/ESCs) have been proposed as a possible source of new photoreceptors to restore vision in these conditions. The proof of concept studies carried out in mouse models of retinal degeneration over the past decade have highlighted several limitations for cell replacement in the retina, such as the low efficiency of cone photoreceptor production from stem cell cultures and the poor integration of grafted cells in the host retina. Current protocols to generate photoreceptors from stem cells are largely based on the use of extracellular factors. Although these factors are essential to induce the retinal progenitor cell (RPC) fate from iPSCs/ESCs, developmental studies have shown that RPCs alter fate output as a function of time (i.e., their temporal identity) to generate the seven major classes of retinal cell types, rather than spatial position. Surprisingly, current stem cell differentiation protocols largely ignore the intrinsic temporal identity of dividing RPCs, which we argue likely explains the low efficiency of cone production in such cultures. In this article, we briefly review the mechanisms regulating temporal identity in RPCs and discuss how they could be exploited to improve cone photoreceptor production for cell replacement therapies.

## Introduction

Retinal degenerative diseases are debilitating disorders that inflict partial or complete loss of vision and are the leading cause of blindness in the developed world. Retinal degenerative diseases are generally characterized by the loss of rod and cone photoreceptors, which are the light-detecting cells of the retina (reviewed in Jones et al., 2017; Tanna et al., 2017). Depending on the disease and genetic mutation involved, rods or cones degenerate first, causing night blindness and tunnel vision or central and daylight vision loss, respectively.

Several approaches such as gene therapy and neuroprotective factors are being explored to prevent or slow down the loss of photoreceptors, but these strategies are generally not restorative, precluding their use in advanced stages of retinal degenerative diseases. In contrast, cell-based therapies offer the potential to restore vision by replacing lost photoreceptors. Endogenous regeneration has been extensively characterized in the teleost retina, where it was shown that Müller glial cells can de-differentiate into progenitor cells and regenerate photoreceptors after retinal injury (Raymond et al., 1988; Cameron and Easter, 1995; Fausett and Goldman, 2006). Studies done in the mammalian retina have highlighted the limitations of endogenous regeneration in higher vertebrates (Ueki et al., 2015; Jorstad et al., 2017), but recent studies have taken advantage of the pioneer factor activity of Ascl1 to trigger Müller glia reprogramming into neurons in the adult retina (Jorstad et al., 2017). However, the majority of the cells produced by reprogrammed Müller cells in these conditions were not photoreceptors and the overall efficiency of regeneration was low.

An alternative avenue to replenish photoreceptors in late stage degenerative retinas is transplantation of stem cell-derived photoreceptors, which might reconnect to the endogenous circuits to restore vision. Pioneering work from various labs has characterized the potential of induced pluripotent stem cells (iPSCs) or embryonic stem cells (ESCs) to differentiate into retinal cells (Zhao et al., 2002; Hirano et al., 2003; Ikeda et al., 2005; Lamba et al., 2006, 2009, 2010; Meyer et al., 2006, 2009; Osakada et al., 2008; Hirami et al., 2009; Eiraku et al., 2011; Tucker et al., 2011; Zhong et al., 2014). As a proof of concept, transplantation studies of rod precursor cells done in mouse models of retinal degeneration have demonstrated some improvement in night-vision-related tasks (MacLaren et al., 2006; Lamba et al., 2009, 2010; Tucker et al., 2011; Pearson et al., 2012; Gonzalez-Cordero et al., 2013). Other studies have focused on transplantation of cone photoreceptors, which were isolated from transgenic donor retinas or ESC-derived retinal organoids expressing GFP from the endogenous promoter of cone genes such as *Chrnb4* and *Ccdc136*, providing proof-of-principle that cone transplantation is also possible (Smiley et al., 2016; Decembrini et al., 2017). The idea of cell transplantation in the retina suffered a set-back recently, however, when it was discovered that the integration of transplanted cells into the retina, if present, is considerably lower than previously interpreted. Transfer of material, including the GFP reporter used to label the transplanted cells, was shown to occur between the transplant and the host retina, leading to over-representation of the engrafted cell population (Pearson et al., 2016; Santos-Ferreira et al., 2016; Decembrini et al., 2017; Ortin-Martinez et al., 2017). In an effort to avoid misinterpreting results due to material transfer, cones were grafted into recipient mice that have lost all photoreceptors, thereby limiting the occurrence of material transfer between the transplanted cone precursors and host photoreceptors in the retina. In these experiments, some engraftment of cones was observed, but it remains unclear to what extent these cells have integrated the endogenous retinal circuits (Smiley et al., 2016; Gonzalez-Cordero et al., 2017; Kruczek et al., 2017). Therefore, it seems clear that more work is required to establish an appropriate approach for cone transplantation in retinal degeneration. Meanwhile, improved protocols for the production of large numbers of cones in stem cell cultures must be developed to meet clinical needs for transplantation.

Cone production in the current protocols of ESC- and iPSC-derived retinal progenitor cells (RPCs) rely on environmental factors, such as Retinoic Acid, Taurine and Notch inhibition, to activate gene combinatorial programs that initiate RPC differentiation into cones (Osakada et al., 2008; Meyer et al., 2009; Lamba et al., 2010; Eiraku et al., 2011; Gonzalez-Cordero et al., 2013; Zhong et al., 2014; Kruczek et al., 2017). Interestingly, it was recently shown that exclusion of retinoic acid signaling is critical for the production of the cone-rich high acuity area in the avian retina (da Silva and Cepko, 2017). Based on these results, it is plausible that even the transient presence of retinoic acid in the stem cell cultures could restrict cone photoreceptor production from the ESC- and iPSC-derived RPCs. In another study, BMP/Wnt/TGFß multifunctional antagonist COCO was found to promote differentiation of sheets of S-opsin+ cone-like cells from hESC (Zhou et al., 2015). Moreover, addition of thyroid hormone induced the switch of S-opsin cone-like sheets to a mixture of S- and M-opsin+ cones from hESCs. It remains unclear, however, whether the cone-like cells promoted by COCO are functional cones and whether the S-opsin+ cell sheets can successfully integrate into a host retina.

But considering what we know from retinal development, it appears unlikely and counter-intuitive that environmental factors alone would be able to induce specific cell fates in stem cell cultures. Indeed, unlike the developing neural tube, where spatial patterning cues play a key part in cell fate decisions, it is well-accepted that RPCs change their potential to generate specific cell types using time rather than spatial positioning (Young, 1985a; Watanabe and Raff, 1990; Cepko et al., 1996; Belliveau and Cepko, 1999; Belliveau et al., 2000). While environmental cues clearly function as feedback inhibition signals to prevent the over-production of specific cell types, the evidence to support an instructive role for environmental cues in promoting cell-fate choice *in vivo* is weak. Conversely, evidence supporting a model whereby RPCs undergo cell-intrinsic changes over time to alter fate output is more convincing. Indeed, heterochronic experiments showed that early- or late-stage RPCs do not change their fate output even when placed in a late or early environment, respectively (Watanabe and Raff, 1990; Belliveau and Cepko, 1999; Belliveau et al., 2000). Additionally, RPCs cultured at clonal density generate a population of clones that is indistinguishable from the clonal population observed *in vivo* (Gomes et al., 2011), even though they develop in an arbitrary culture medium that has little resemblance to the *in vivo* environment. The general birth order is also maintained in such cultures, arguing in favor of an intrinsic program operating in RPCs to control fate output. Whether these programs could be exploited to favor the production of specific retinal cell types in ESC and iPSC cultures remains unexplored. We discuss this idea below.

## Temporal Patterning in the Retina

The most immature RPCs have the competence to generate all seven cell types of the retina (Agathocleous and Harris, 2009; Bassett and Wallace, 2012; Cepko, 2014; Brzezinski and Reh, 2015), but do so in an overlapping chronological order. Early in development, they generate mostly early-born cell types like ganglion, horizontal, cone and amacrine cells, and then transition to generate mostly late-born cells like rods, bipolar, and Müller glia at later stages of development. As mentioned above, RPCs rely largely on intrinsic factors to control their “temporal identity”, a period during which they are able to give rise to only a specific subset of cell types. This concept of temporal progression of cell fate output was first suggested in what is now widely referred to as the “competence model” (Watanabe and Raff, 1990; Cepko et al., 1996). But the specific factors instructing temporal identity in RPCs have remained largely elusive until recently.

Temporal progression in neural progenitors was extensively studied in the *Drosophila* central nervous system, where the sequential expression of temporal identity factors like *hunchback>kruppel>pdm>castor* control the order of neurons produced in neuroblast lineages (Isshiki et al., 2001; Pearson and Doe, 2003; Tran and Doe, 2008). Another temporal cascade consisting of *homothorax>eyeless>sloppy-paired1/2>dichaete>tailless* transcription factors functions similarly in the *Drosophila* optic lobe (Li et al., 2013). A follow-up study demonstrated that spatial cues in the D/V axis incur specific differences in the lineages generated by these intrinsic temporal cues in the optic lobe, suggesting the collaboration of spatial and temporal factors in the production of neuronal diversity (Erclik et al., 2017). Intrinsically, the crosstalk and feedback inhibition of these factors allows transition from the expression of one temporal factor to another (Pearson and Doe, 2003; Tran and Doe, 2008). Similarly, in the murine retina, Ikaros (*Ikzf1*), the mammalian ortholog of *hunchback*, confers the ability of RPCs to generate early born cell fates such as amacrine, horizontal and ganglion cells (Elliott et al., 2008). On the other hand, *Casz1*, the mammalian ortholog of *castor*, grants RPCs the potential to generate late born cell types such as rods and bipolar cells (Mattar et al., 2015). Importantly, temporal factors endow the competence to generate specific combination of cell types in progenitors, rather than controlling the timing of cell cycle exit. Mechanistically, Ikzf1 represses Casz1 expression in the early RPCs, whereas Casz1 is de-repressed by the loss of Ikzf1 in late progenitors. These factors are both necessary and sufficient to drive their respective cell fates in the developing retina, much like they are in *Drosophila* neuroblast lineages, suggesting a conservation of the temporal cascade from invertebrates to vertebrates. Interestingly, Ikzf1 also contributes to the establishment of the temporally restricted cell fates in the developing mouse neocortex (Alsiö et al., 2013), suggesting that Ikzf1 might have a role as an intrinsic temporal identity factor in other progenitor contexts. How exactly Ikzf1 functions to regulate temporal identity remains unknown, but work in lymphocytes showed that Ikzf1 can function as a chromatin accessibility factor (Kim et al., 1999). It is therefore tempting to speculate that Ikzf1 could function by closing critical regions of the RPC chromatin required for the expression of genes involved in late-born cell type production.

## Rod and Cone Photoreceptor Production: A Temporal Identity Crisis

As mentioned above, cone photoreceptors are generated during early stages of retinal development, whereas rods are mostly produced late. While cones can arise from multipotent RPCs, there is also evidence that cones are produced from lineage-restricted RPCs. During early retinogenesis when cone production peaks, RPCs expressing the bHLH transcription factor Olig2 are strongly biased to produce a terminal division containing at least one cone, whereas at post-natal stages Olig2-positive RPCs are biased to producing rods (Hafler et al., 2012). Thus, Olig2-derived lineages generate different cell types depending on the temporal window of retinogenesis, suggesting that the transition from early to late Olig2^+^ lineages might be governed by temporal identity factors, although this is yet to be explored. Nonetheless, the transition from cone to rod production in the embryonic and post-natal retina could partly be explained by the activation of downstream effectors that initiate rod and suppress cone genesis, or vice versa.

A proposed mechanism for the cone/rod switch is the transcriptional dominance model. RPCs initially divide and commit to a generic post-mitotic photoreceptor precursor in response to upstream signaling events that remain unclear (Swaroop et al., 2010). During the peak of cone genesis, the expression of Nrl, a rod specification factor, is present in only a subset of photoreceptor precursors, which become rods, whereas the remaining majority do not express Nrl and become cones (Young, 1985a,b; Mears et al., 2001; Akimoto et al., 2006). Based on these results, it was proposed that generic photoreceptor precursors are destined to become cones by default, and require the activity of Nrl to generate rods. As the retina transitions to post-natal stages, Nrl is activated in most, if not all photoreceptor precursors to trigger the rod fate via induction of Nr2e3 expression, a nuclear receptor involved in suppressing cone genes (Oh et al., 2008). Therefore, the transcriptional dominance model could be applied to both fate-restricted and multipotent RPCs that divide to produce generic photoreceptor precursors. A proposed model of transition from cone-producing to rod-producing phase in the lineage-restricted RPC population is that Olig2^+^ embryonic RPCs exit the cell cycle to first give rise to generic photoreceptor precursors that have minimal expression of Nrl, thereby biasing the differentiation of precursors into cones. Once the expression of Nrl elevates at postnatal stages, the generic photoreceptor precursors are pushed towards the rod fate. According to this model, Nrl acts as the upstream fate switch for cone/rod specification and, in principle, this mechanism could be manipulated to specifically promote cones in ESC-derived RPCs. However, while inactivation of Nrl generate S-cone-like cells, they lack key characteristics of actual cones, displaying stunted outer segments and aberrant expression of some rod genes (Daniele et al., 2005; Montana et al., 2013).

While Nrl appear to function by repressing the cone fate, there is also evidence of cone-promoting transcription factors. Emerson et al. (2013) investigated the transcriptional regulation of the thyroid hormone receptor beta (*thrb*) gene, one of the earliest marker of cones (Ng et al., 2001, 2009). Cis-regulatory modules (CRMs) of *thrb* are regulated by the co-operation of Onecut1, a cut homeobox transcription factor and Otx2, a homeobox transcription factor necessary for photoreceptor production and maintenance (Nishida et al., 2003; Koike et al., 2007; Sato et al., 2007). Onecut1 is initially expressed in Olig2^+^-early RPCs when they are required for the specification of cones and horizontal cells (Emerson et al., 2013; Sapkota et al., 2014). Onecut1 is transiently induced in Otx2^+^ precursors that differentiate into cones, whereas it is sustained in the precursors lacking Otx2 expression that become horizontal cells. Importantly, Onecut1 is sufficient to induce cone production outside their normal temporal window, as evidenced by the misexpression of Onecut1 in post-natal RPCs, which leads to the production of Rxrγ-expressing cone-like cells (Emerson et al., 2013). Since one of the largest hurdles of cell replacement therapies has been to specifically promote cones in the ESC-derived retinal progenitors, one might think that induction of Onecut1 expression would be a promising approach to induce cone production for transplantation purposes. However, cones generated by sustained Onecut1 misexpression are immature, as they solely express genes required for the early specification and not the genes necessary for photo-transduction and functionality (Emerson et al., 2013). Transient activation of Onecut1 could potentially favor the production of mature cones expressing late genes but this has not been tested. Another avenue could be to express Onecut1 together with Sall3, a transcription factor sufficient and necessary for the induction of late S-cone specific genes (de Melo et al., 2011), which might produce functional cones that could be ideal for transplantation, but this remains to be tested. Alternatively, the activation of a more upstream pathway in RPCs may be required to promote the efficient production of fully mature and functional cones.

## Temporal Reprogramming to Increase Cone Photoreceptor Production

**Figure 1:**
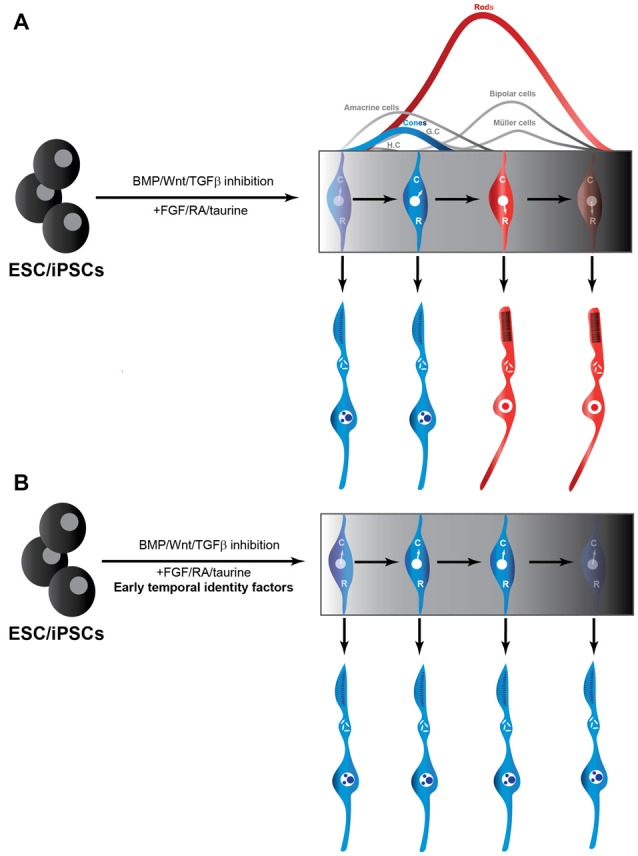
Production of cones from iPSC/ESCs may be facilitated by temporal identity factors. **(A)** Traditional use of extrinsic cues to promote RPCs from ESCs and iPSCs. RPCs through their intrinsic developmental program to initially generate cones (C) and transition into a rod (R)-producing phase at later stages in the culture (Rapaport et al., 2004). **(B)** Addition of temporal factors to established protocols for producing RPCs from ESCs and iPSCs could be exploited to freeze the RPCs in a cone-producing phase. RPC, retinal progenitor cells; ESC, embryonic stem cells; iPSC, induced pluripotent stem cell.

Given that RPCs undergo cell-intrinsic changes in their competence to generate cones, as discussed above, we posit that these temporal transitions also operate in ESC-derived RPC cultures. As a result, RPCs rapidly lose the competence to generate cones and instead acquire the competence to generate rods, which might explain why production of rods is more efficient than that of cones in such cultures. We argue that manipulating the temporal identity of RPCs will be critical to improve cone production from ESCs, by prolonging the cone-production window (Figure 1). Since temporal factors do not force the RPCs to exit the cell cycle, they could also potentially improve cone production capacity of ESC-derived RPCs by halting the progression of the RPC temporal state, without restricting the pool of available progenitors. Moreover, by induction of early temporal factors, it might be possible to revert RPCs with a late temporal identity to an earlier stage during which they are competent to produce cones. Knocking down late temporal factors could also potentially prevent the progression into late temporal identity windows, and revert RPCs back to the cone-producing state. Consistent with this idea, inactivation of the late temporal identity factor Casz1 increased cone production in the developing mouse retina, whereas ectopic expression during early stages reduced cone genesis (Mattar et al., 2015). In other words, temporal factors could provide a way to “freeze” the temporal identity of RPCs at will to achieve the desired fate output, without scaling up the number of costly ESC cultures. Most importantly, the late genes required for photo-transduction and maintenance are predicted to be expressed in cones derived from manipulation of temporal identity factors. Indeed, since a potential mode of action of temporal factors is by modulating chromatin accessibility (Kim et al., 1999), they might allow efficient activation of the full set of genes required for the generation of functional cones, without forcing the RPCs to exit the cell cycle. A potential limitation of temporal factors, however, could be that they might also promote the generation of other cell fates produced during the early temporal window such as ganglion, horizontal and amacrine cells, as was found in misexpression experiments *in vivo* (Elliott et al., 2008). Since there are currently no known temporal identity factors required for cone production specifically, it will be interesting to explore other mammalian orthologs of the members of the *Drosophila* temporal cascades.

## Conclusion and Perspectives

While we focused here on the production of cone photoreceptors, another important hurdle is to find ways to improve integration efficiency of transplanted photoreceptors into the host retinas. Ultimately, approaches that would favor both cone production and integration would be ideal. It is tempting to speculate that manipulating temporal identity might also promote integration by conferring an immature and perhaps more migratory phenotype to the grafted cells. Future experiments should help test these and other ideas to replace cone photoreceptors, as this will have major implications in the development of therapies for retinal dystrophies.

## Author Contributions

AJ and MC wrote the manuscript.

## Conflict of Interest Statement

The authors declare that the research was conducted in the absence of any commercial or financial relationships that could be construed as a potential conflict of interest.

## Abbreviations

CRM: cis-regulatory modules
ESC: embryonic stem cells
iPSC: induced pluripotent stem cell
RPC: retinal progenitor cells.
